# Mitigating the Rising Burden of Non-Communicable Diseases through Locally Generated Evidence-Lessons from Tanzania

**DOI:** 10.5334/aogh.4111

**Published:** 2023-11-17

**Authors:** Harrieth P. Ndumwa, Davis E. Amani, Jackline E. Ngowi, Belinda J. Njiro, Castory Munishi, Erick A. Mboya, Doreen Mloka, Amani I. Kikula, Emmanuel Balandya, Paschal Ruggajo, Anna T. Kessy, Emilia Kitambala, Ntuli Kapologwe, James T. Kengia, James Kiologwe, Omary Ubuguyu, Bakari Salum, Appolinary Kamuhabwa, Kaushik Ramaiya, Bruno F. Sunguya

**Affiliations:** 1 Muhimbili University of Health and Allied Sciences, 9 United Nations Road, Upanga West P O Box 65001, Dar es salaam, Tanzania; 2 Ministry of Health, P O Box 743, Dodoma, Tanzania; 3 President’s Office Regional Administration and Local Government, P O Box, 1923 Dodoma, Tanzania; 4 Tanzania Non-Communicable Diseases Alliance, P O Box 65201, Dar es salaam, Tanzania; 5 Tanzania Diabetes Association, P O Box 65201, Dar es salaam, Tanzania; 6 Shree Hindu Mandal Hospital, P O Box 581, Dar es salaam, Tanzania

**Keywords:** Non-communicable diseases (NCDs), epidemiological transition, advocacy, multi-stakeholders, Tanzania

## Abstract

**Background:**

The burden of Non-Communicable Diseases (NCDs) is rapidly increasing globally, and low- and middle-income countries (LMICs) bear the brunt of it. Tanzania is no exception. Addressing the rising burden of NCDs in this context calls for renewed efforts and commitment by various stakeholders. This paper highlights local initiatives and strategies to combat NCDs in Tanzania and provides lessons for countries with similar contexts.

**Methods:**

We reviewed published and grey literature and conducted policy analysis on NCDs in Tanzania to examine the burden of NCDs and the national response addressing it. The documents included National NCD strategic plans, NCD research agenda, and reports from the World Diabetes Foundation and the World Health Organization. Moreover, a scoping review of ongoing NCD activities and programs in other countries was also conducted to supplement the evidence gathered.

**Results:**

The rising burden of NCDs as a result of the epidemiological transition in Tanzania called for the launching of a dedicated National NCD Control and Prevention Program. The Ministry of Health collaborates with local, national, and international partners on NCD prevention and curative strategies. This led to the development of important guidelines and policies on NCDs, including strengthening the capacity of health facilities and healthcare workers, increased community engagement and awareness of NCDs, and increased advocacy for more resources in NCD initiatives. Strong governmental commitment has been vital; this is demonstrated by a renewed commitment to the fight through national NCD week and related advocacy activities conducted annually. To ensure multi-stakeholders’ engagement and political commitment, all these activities are coordinated at the Prime Minister’s office and provide strong lessons for countries with contexts similar to Tanzania.

**Conclusion:**

Multi-stakeholders’ engagement, innovative approaches, and coordinated governmental efforts to address NCDs have shone a light on addressing the burden of NCDs and may be sustainable if aligned with locally available resources. Such initiatives are recommended for adoption by other nations to address the burdens of NCDs.

## Background

The burden of Non-Communicable Diseases (NCDs) has rapidly increased globally, making NCDs a leading cause of disability, morbidity, and mortality [1]. They accounted for about 41 million deaths globally—close to three in every four deaths [2]. About 17 million people die from NCDs at the most productive age, with eight in ten of such deaths occurring in LMICs [2]. While NCD mortality is declining in high-income countries, the epidemiological transition seems to take a toll on low- and middle-income countries (LMICs), which now harbor 85% of NCD-related deaths [2]. Diabetes mellitus, cancers, cardiovascular diseases, and chronic respiratory diseases account for about 80% of these deaths [3,4]. The resulting burden is also associated with nutrition, demographic, and economic transitions in LMICs [3].

Tanzania, like other LMICs, is experiencing the fast-growing burden of NCDs in the context of persistent communicable diseases and a relatively weak health system. The big four NCDs globally (cardiovascular diseases, cancers, chronic respiratory diseases, and diabetes) contribute to about 40% of the NCD burden in Tanzania [5]. Different from the global picture, the common four risk factors can only explain less than half of the NCD burden in the country, calling for more local evidence and therefore a tailored approach to NCD prevention. The most common conditions in Tanzania, hypertension and diabetes, were prevalent among 25.9% and 9.1% of the population respectively, as found by a recent nationally representative survey [6]. Other conditions include cardiovascular diseases, cancers, chronic respiratory diseases, oral diseases, mental illnesses and substance abuse, accidents, and injuries [7]. While about one-third of all deaths are currently due to NCDs, the burden is projected to overtake all communicable diseases combined [7].

The rapidly growing burden of NCDs in the context of traditionally persistent communicable disease conditions in Tanzania has weakened health systems, drained the available resources, and put tremendous pressure on individual and national economies [8]. As a result, access to important preventive, curative, and rehabilitative services has remained a struggle, resulting in higher morbidity and mortality rates, particularly among the under-served younger populations who often lack access to quality health services in relation to their actual health needs [9]. This calls for evidence-based strategies to inform policies, practice, and members of the community to join efforts to address NCDs.

In responding to the increasing NCD burden, the government of Tanzania has strived to strengthen access to quality health services, policies, and strategies informed by local evidence on NCDs. In collaboration with other stakeholders, the government has established and implemented initiatives to curb this unprecedented threat to national development. Through this process, it is important to take stock of milestones, the evolution of strategies, and the implementation of interventions and initiatives aiming to address the burden of NCDs in Tanzania. This will help to inform the progress of the campaign and draw lessons for other countries with a similar context.

## Methods

**Design and context:** As a policy paper, this article mainly involved policy analysis and a desk review of both published and grey literature exploring the evidence available on NCD programs and initiatives in Tanzania. These helped to highlight the initiatives and milestones attained since the launch of the NCD Control and Prevention Program in Tanzania, a first in the region, aiming to coordinate national efforts to address the burden of NCDs.

**Review process and data abstraction:** We reviewed both published and grey literature that have described the burden of NCDs, National NCD program historical milestones, NCD program mandates and activities, and NCD advocacy activities. A thorough review and documentation of the engagement of civil societies and other stakeholders in the fight against NCDs was conducted. Moreover, we reviewed and documented challenges and opportunities facing the execution of set plans and strategies for NCDs in Tanzania. The documents included: the NCD strategic plan 2016–20; the National NCD Research Agenda 2022; reports from civil societies and the World Diabetes Foundation (WDF); and the Ministry of Health’s reports on various NCD activities in the country. Similar reports by the World Health Organization and other regional and international bodies were also reviewed. The team conducted a narrative synthesis of the gathered literature to compile evidence of the progress and lessons learned that can inform the government and other countries with similar contexts.

**Ethical consideration:** This evaluation did not require ethical approval owing to the nature of the evidence used. The evaluation team consisted of scientists and researchers from the Muhimbili University of Health and Allied Sciences, the Tanzania Diabetes Association, the Ministry of Health, the President’s Office- Regional Administration and Local Government, and other implementing partners.

**Patient and public involvement:** No patient was involved.

## Findings

### The burden of NCDs in Tanzania

Evidence shows that in Tanzania, NCDs have the highest contribution to the Disability Adjusted Life Years (DALYs) across all age groups, and the magnitude is higher among the older population [5]. The burden of NCDs has increased remarkedly over the past 30 years in the country. DALYs as a result of NCDs in Tanzania have doubled from 18.28% in 1990 to 36.39% in 2019 [10].

In accordance with the available nationally representative survey and isolated evidence from locally conducted studies, the most common NCDs in Tanzania are cardiovascular diseases (25.9%), diabetes (9.1%), chronic respiratory diseases (17.5%), and mental health conditions (11%) [5,6,11]. NCDs contribute to nearly a third of all deaths in the country [7]. Cardiovascular conditions (30–40%), cancers (18.6%), COPDs (18.4%), and injuries (17.9%) account for the largest proportion of all mortalities due to NCDs [12]. The overall rate of mortality from NCDs has increased by 153.3% between the years 2006 and 2015; four in every ten such deaths occurred at regional referral hospitals, and about a third occurred at primary-level facilities [12]. Evidence estimates the country has about 40,464 new cases of cancer every year [13]. In terms of burdens, the most common cancers are cervical (25%), breast (10%), prostate (8.8%), and oesophageal (6.5%) [13]. Late diagnosis and presentation, as well as a limited number of facilities available for cancer management remain a barrier to addressing the burden of cancer in the country. Despite the increase of NCDs, other conditions such as infectious diseases, neonatal and maternal conditions, and nutritional diseases still remain a challenge, so the country experience a double burden of disease [10].

The burden of NCDs has also financially strained families and societies. Evidence suggests that the prevalence of catastrophic spending and impoverishment is high among households with NCDs [14]. The odds of households experiencing catastrophic health spending are 52.7% higher when there is a family member with NCD, as compared to households without one [14]. The burden of NCDs is also straining the health financing system, as evidenced by the reimbursement of the National Health Insurance Funds (NHIF). The Tanzanian NHIF Report in 2022 shows that only three NCD services—namely haemodialysis, chemotherapy, and care and treatment for cardiovascular diseases—accounted for 20% of all the collected funds in 2021.

NCDs account for most hospitalizations, DALYs, and nearly three-quarters of global mortality [15,16]. The Sustainable Development Goals target 3.4 aims to reduce by one-third premature mortality from non-communicable diseases through prevention and treatment and promote mental health and well-being by 2030 [17]. High-level meetings of the United Nations General Assembly emphasize the role and importance of multi-stakeholders’ engagement in realizing these targets [18]. The political declaration of the 2011 United Nations High-Level Meeting (UNHLM) on the prevention and control of NCDs recognized the role of civil societies in supporting national efforts geared to address the burden of these diseases. It called for strengthening the coordination of these stakeholders in order to improve effectiveness.

### Evolution of the national NCD program

**Figure 1:**
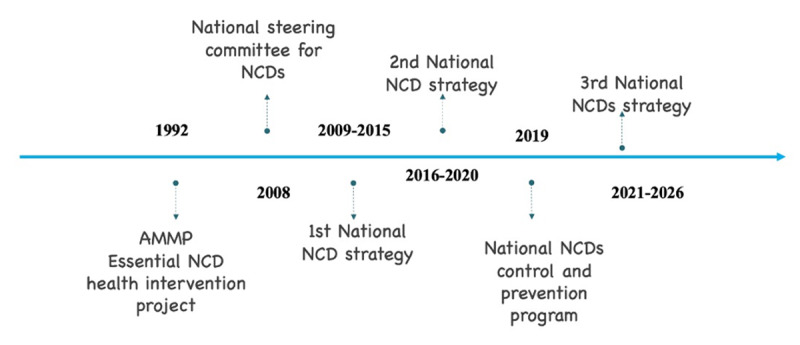
NCD program historical milestones in Tanzania.

Tanzania promoted the integration of civil societies (CSOs) and other non-governmental organizations (NGOs) to accelerate the development and execution of plans and strategies geared towards address the rising burden of NCDs. The local response to NCDs in Tanzania began more than three decades ago with the activities of the Adult Morbidity and Mortality Projects (AMMP) and others, such as the essential NCD Health Intervention Project (Figure 1) [20]. The AMMP was established in 1992 by the then-Muhimbili University College of Health Sciences (MUCHS), the Ministry of Health of Tanzania (MoH), and the University of Newcastle in the United Kingdom. The purpose was to conduct a surveillance system that would provide cause-specific death rates in a three-year duration among adults and to link community-based surveillance to evidence-based planning for health care [20].

The Ministry of Health (MoH) in Tanzania, in collaboration with the Tanzania Non-Communicable Diseases Alliance (TANCDA), Tanzania Diabetes Association (TDA), and other stakeholders, pioneered the development of a National Strategic Plan for NCDs and the development of the National NCD Control and Prevention Program. The program is centrally coordinated at MoH and was launched by Hon. Kasim Majaliwa Majaliwa (MP), the Prime Minister of the United Republic of Tanzania in November 2019 [19]. This marked the official operationalization of the strategy and the implementation of actions against NCDs, coordinated by the Prime Minister’s office to ensure multi-sectoral engagement.

In 2008, the then-Ministry of Health and Social Welfare established the NCD division to help develop policies and monitor progress in response to NCDs and injuries. The division is currently working to strengthen the capacity for both the prevention and management of NCDs and has a functional National Steering Committee for NCDs (Figure 1) [21]. The first National NCD Strategy for the prevention of NCDs was launched in 2009, covering the period between 2009 and 2015. It was based on the WHO strategic framework and the Global Strategy on diet and physical activities [7]. This was subsequently followed by the SECOND NCD MULTI-SECTOR STRATEGY AND ACTION PLAN OF 2016–2020, which expanded to include further preventive and rehabilitative services. In November 2016, the national Non-communicable Diseases and Injury (NCDI) poverty committee was established. The committee was focused on priority setting for NCDs in settings of poverty, and it functions as a sub-group of the existing multisectoral NCD group in the Ministry of Health. The third strategic plan was proposed for the period of 2021–2026 through a participatory process guided by the Ministry of Health. From 17 to 18 September 2019, a framework for multisectoral collaboration, consisting of relevant stakeholders, was initiated in Dar es Salaam, Tanzania; it involved more than 100 stakeholders from the government, NGOs, research institutions, religious associations, law institutions, and academia [22]. The evolution of the Tanzanian National NCD program over the past three decades has each time made improvements that cut across all aspects of NCDs, from prevention, to treatment and rehabilitation. Multi-stakeholder’s engagement has been consistent throughout all phases and has been vital in ensuring that NCDs are addressed in totality, while affordable and quality care is maintained.

### NCDs program mandates and activities

The National NCD prevention and control program under the Ministry of Health is the leading implementing body for NCD-related interventions in Tanzania. The program has been working in line with the guidance provided by the National Policies, Sectoral Strategic Plans, the United Nations Political Declaration on the Prevention and Control of NCDs, and the Global Action Plan for the Prevention and Control of NCDs [18,23]. Since its establishment, the aim has been: to support all issues related to governance and coordination; to identify priority areas for intervention and research; resource mobilization; and the coordination of key stakeholders, as well as service provision, especially at district and regional hospitals in the country. However, efforts are being made to ensure that basic services, such as screening for NCDs, are readily available in primary healthcare facilities—which are noted to have limited basic diagnostic equipment and staff trained to treat NCDs [24,25]. Thus, the National NCD prevention and control program has set out different strategies to address NCDs in the country, as described in Table 1 below.

**Table 1:** Strategies and approaches to address NCDs in Tanzania.


STRATEGY	DESCRIPTION

*Multi-stakeholder engagement*	Collaboration with local and international stakeholders: the Non-Communicable Diseases Alliance (TANCDA), Tanzania Diabetes Association (TDA), World Health Organization (WHO), Tanzania Cancer Association (TCA), Tanzania Association for Respiratory Diseases (TARD), Heart Foundation of Tanzania (HFT), World Diabetes Foundation (WDF), and institutions such as the Tanzania Food and Nutrition Centre (TFNC), Tanzania Food and Drug Authority (TFDA), and the Tanzania Bureau of Standards (TBS).

	Crossing beyond health systems to engaging other sectors, including education, agriculture, communication, employment, energy, environment, finance, food. Also establishing multisectoral committees at different administrative levels, acknowledging that many determinants of NCDs are outside the health sector.

*Development of guidelines and policies*	Working with scientists, policy makers, health personnel, and other partners to develop an NCD strategic plan, which is now being relied upon to develop and implement plans and strategies addressing the burden of NCDs in the country.

	Advocacy, treatment, and care guidelines for specific diseases, such as sickle cell disease (SCD), hypertension, stroke, and diabetes mellitus have been developed, used for the routine care of patients with NCDs.

	The National NCD Research Agenda has been developed, stipulating the research areas of national interest, also serving as an important guide to scientists and potential funders to select priority areas for NCD research.

*Strengthened capacity of health facilities and health care workers*	In collaboration with TDA and TANCDA, the NCD unit has managed to establish over 150 clinics throughout the country, which are all supplied with diagnostic guidelines and the equipment necessary for the management of NCDs.

	Hundreds of healthcare workers from Northern Tanzania, the Lake Zone, and other parts of Tanzania have been trained on NCD prevention, diagnosis, and treatment through support from DANIDA and WDF, in collaboration with the civil society organizations.

*Increased community engagement and awareness on NCDs*	Campaigns that focus on prevention and fostering good health-seeking behaviors, which will help ensure timely diagnosis and management of NCDs; these campaigns have been conducted through radio, TV sessions and physical seminars.

	One hundred thousand school children have been provided with basic knowledge on risk factors for NCDs and have undergone screening for some of the NCDs.

	Over six million individuals were reached by outreach screening and sensitization campaigns conducted in the Lake Zone and Northern Tanzania


### The National NCD research agenda

WHO recommends high-quality research and development to enable evidence-based NCD responses [26]. However, only a handful studies on NCDs have been conducted in LMICs, and even fewer are from countries with a fast growing burden of NCDs like Tanzania [27]. In this regard, the National Strategic Plan for NCDs and the National NCD Prevention and Control Program have highlighted research as one of the core elements of the National NCD response. To further underline the importance of research on NCDs, the first National NCD research agenda was developed by the National NCD Prevention and Control Program, involving research and academic institutions in January 2022. The research agenda aligns with the fourth objective of the Sixty-first World Health Assembly, which endorsed the Global Strategy Action Plan for the Prevention and Control of Non-communicable Diseases. Its aim is to *“promote research for the prevention and control of non-communicable diseases.”* [28,29]. Other countries have also developed and are operationalizing their own NCD research agendas [30]. The national research agenda for NCDs aims to prioritize areas where the government and implementing partners need evidence for an NCD response. Moreover, it is used by different local and international stakeholders to increase their understanding of Tanzania’s research context and the elements that should be considered for funding and conducting basic health system, clinical, and implementation research.

### NCD advocacy activities

The national NCD week in Tanzania was initiated in 2019, along with other NCD initiatives. To date, three NCD week activities have been implemented, one each year covering all regions. In the first year (2019), the national event for NCD week was commemorated in the capital city, Dodoma. This was followed by Dar es Salaam, the business capital with the biggest burden of NCDs in 2020. In 2021, the national commemorations were held in Arusha, the tourism hub of the country. Apart from the active involvement of the government in the advocacy services for NCDs, the private sector and religious bodies have also assumed active roles in raising awareness of NCDs, alerting populations of the NCD services available, in addition to advocating for multi-stakeholders’ involvement. Main recommendations have been physical activities, such as sports and jogging; NCD exhibitions; awareness and education on NCDs; NCD screening and linkage to care; and NCD advocacy in schools and media [31]. The NCD weeks have been Climax by the National NCD Scientific Conferences, creating a platform for stakeholders to discuss and disseminate local NCD research findings, analyze the NCD situation, and discuss solutions to address the NCD burden in the country. These efforts are in line with the Global Week for Action on NCDs, which was first organized in 2018; this mainly unites the NCD movement each year under a specific theme to combine efforts in reducing the global burden of NCDs [32].

### Analysis of key policy documents

The policy analysis of non-communicable disease (NCD) documents in Tanzania provides a comprehensive overview of the country’s efforts to address the burden of NCDs. The policy analysis in this paper focus on four key policy documents, including the Tanzania Steps Surveys Report (2013), the Strategic and Action Plan for the Prevention and Control of NCDs in Tanzania 2016–2020, the Tanzania Non-Communicable Diseases and Injuries Poverty Commission (2020), and the National NCD Research Agenda (2022).

*The Tanzania Steps Surveys Report (2013)* [6] presents data on NCD risk factors, including tobacco use, physical inactivity, an unhealthy diet, alcohol consumption, and also highlights the prevalence of NCD conditions, such as obesity, high blood pressure, and diabetes. It helps to identify high-risk populations and informs targeted interventions for NCD prevention and control.

*The Strategic and Action Plan for the Prevention and Control of NCDs in Tanzania 2016–2020* [7] serves as a roadmap for NCD prevention and control in Tanzania. It outlines strategic goals, objectives, and interventions aimed at reducing the impact of NCDs on the population. The plan emphasizes the importance of multi-sectoral collaboration, health promotion, and community engagement to tackle NCD risk factors and improve healthcare services for those affected by NCDs.

*The Tanzania Non-Communicable Diseases and Injuries Poverty Commission (2020)* [5] focuses on addressing the social and economic impacts of non-communicable diseases and injuries (NCDIs). It provides details on the relationship between poverty and NCDIs and proposes strategies to reduce the burden of NCDIs on vulnerable populations. The report documents an assessment of the readiness of the health system to respond to NCDIs in order to further propose cost-effective interventions for priority NCDI conditions. The report also includes a set of proposed priority NCDI interventions and the proposed estimates of the investment required for implementation.

*The National NCD Research Agenda (2022)* [19] highlights research priorities related to NCDs in Tanzania. It involves analyzed evidence from local and global research that have conducted situational analysis and hence, developed NCD research themes. These highlight existing gaps to address the burden of NCDs and propose priority research areas. The six key themes highlighted in this research agenda include: the burden of NCDs; risk factors for NCDs; enabling environments; health systems; innovative and implementation research for NCDs; and multisectoral approaches in addressing NCDs and their related comorbidities. The agenda emphasizes the need for evidence-based interventions and the translation of research findings into policy and practice.

## Discussion

Tanzania represents many of the countries in sub-Saharan Africa with an increasingly growing burden of NCDs—attributed to demographic transition characterized by: an ageing population; economic transition characterized by urbanization, sedentary lifestyles, and the changing nature of economic activities; and nutrition transition characterized by unhealthy dietary patterns, low physical activity thresholds, and therefore overweight and obese populations [33]. The total DALYs due to NCDs in the sub-Saharan African have increased by about 70% between 1990 and 2009 [33]. While NCDs have remained the leading cause of morbidity and mortality in high-income countries, they are on a slow decline compared to the situation of LMICs [34]. The burden is rising fast in Tanzania and is projected to overtake the burdens of traditionally prevalent communicable diseases within a decade. Such a burden has already strained the struggling health system, challenging healthcare financing, and subjecting societies, families, and individuals to poverty.

Addressing the impending NCD threat to national development called for strengthened policies and strategic plans in Tanzania. The development of the NCD National Strategic Plans and the NCD Control and Prevention Program under the Ministries of Health is also seen in other countries such as Kenya [35], India [36], Norway [37] and across different WHO regional offices [38]. A strong collaboration between Ministries of Health, non-governmental organizations (NGOs) and Civil Society Organizations (CSOs) have added value in addressing the burden of NCDs in Tanzania, similar to other countries with stronger health systems and economies [39]. Moreover, the WHO recognizes the relevance and value of a sustained and strong civil society organization (CSO) voice in bringing solutions to address NCDs and has created the WHO Civil Society Working Group (CSWG) on Noncommunicable Diseases (NCDs) [40]. This paper has outlined some of the NCDsprograms and activities in Tanzania, along with their corresponding successes, with the main ones being multi-stakeholders’ engagement, development of guidelines and polices, strengthened capacity for HCWs on NCDs, and increased community engagement and awareness of NCDs. Similar initiatives have been carried out in other countries and have also been shown to have an impact towards addressing NCDs. Multi-stakeholder, multisectoral collaboration is considered as a crucial component in the implementation of NCD policies in every country [41]. Moreover, countries such as South Africa have also established evidence on the multiple roles of healthcare workers, particularly those working in community levels, in responding to the raising burden of NCDs [42]. In Mozambique, improved training and supervision for healthcare workers has helped in preventing and controlling cardiovascular diseases at primary healthcare levels, hence improving national NCD care delivery [43]. In the strategy to achieve universal health coverage, primary healthcare for NCDs is also provided among marginalized populations, such as refugees and healthcare workers that have been on the frontline [44].

Community engagement and participation are reported to be important elements in strengthening preventive measures towards NCD prevention in LMICs [45,46]. In southeastern Nigeria, community engagement has been demonstrated to contribute towards long-term sustainability in improving the management of NCDs [47]. With the current emphasis on people-centered health care and universal health coverage [26], the NCD prevention and control program in Tanzania continues to actively engage community health workers, health management teams at district and referral levels, community leaders, schools, the media, and other stakeholders in NCD prevention, health promotion and awareness activities [22], a practice that is also noted in many other countries [35,36,37,39,42,47].

The implementation of NCD program activities in Tanzania still faces several challenges. Financial constraints limit the scope of these activities and thus threaten the sustainability of the gains made. Human resource for health is still the major outcry in Tanzania for both NCD and communicable disease interventions [48]. This, in addition to limited availability, affordability, and accessibility of other resources, such as diagnostics and drugs, jeopardize the overall quality of healthcare rendered to patients. Low research output denies policy makers and other stakeholders the evidence necessary to develop and revise policies and strategies for NCDs. Moreover, the double burden of diseases due to coexisting communicable diseases, as well as emerging and re-emerging diseases, including COVID-19. poses a threat to effectively address NCDs in the country [49].

Despite the challenges, a number of opportunities have been realized to address them. A successful establishment of stakeholders’ framework for NCDs provides the NCD Program with an excellent platform to discuss and implement health promotion and prevention services. The unit can also leverage on the framework to seek and introduce innovative financing models for NCDs, which will help address resource limitations that characterize our health systems. To a great extent, the success of HIV/AIDS and TB programs have been aided by the engagement of community health workers and the introduction of community-based promotion, prevention, testing, and treatment programs [50]. The integration of NCD advocacy strategies into these existing programs could be a game changer, especially now that a lack of resources, particularly skilled human resources, remain a major health system crisis. Collaborating with the Ministry of Education to increase students’ enrollment into health carders and fostering the development and inclusion of NCD-related teaching programs will contribute to decreasing the human resource for health crises. Advancements in science and technology in many parts of the world have been integral in enhancing the response and resilience of healthcare systems [51]. Tapping into these developments, especially in a transitioning era of disease epidemiology, is necessary to promote risk communication, and to enhance the efficiency and effectiveness of the strategies being undertaken.

Moreover, a brief analysis of key policy documents, including the Tanzania Steps Surveys Report (2013), the Strategic and Action Plan for the Prevention and Control of NCDs in Tanzania 2016–2020, the Tanzania Non-Communicable Diseases and Injuries Poverty Commission (2020), and the National NCD Research Agenda (2022) provided valuable insights into the strategic direction, research priorities, and the challenges of addressing NCDs in Tanzania. The findings contribute to evidence-informed decision making and serve as a foundation for future research and policy development in the field of NCD prevention and control in Tanzania.

## Conclusion

The burden of NCDs is growing at an alarming speed in Tanzania, similar to other LMICs. Despite various challenges, through the Ministry of Health, Tanzania has demonstrated outstanding efforts and commitment to address this growing threat. Various programs and activities have been implemented from the community to national level, including the establishment of NCD units under the Ministry of Health, NCD strategic plans, National NCD research agendas, and a number of NCDs advocacy activities. Understanding that multiple and crosscutting efforts are needed to address NCDs, we call for countries with similar contexts (in terms of NCD burden and characteristics) to implement such efforts, in particular establishing and strengthening NCD units within the Ministries of Health to ensure central and effective coordination of NCD activities. Moreover, the realization of stakeholders’ engagement in addressing the raising burden of NCDs has been key in the success of NCD activities. Collaborations with academic institutions and NGOs to build capacity, strengthen, and generate evidence-based research on NCDs, and developing policies and strategies to address NCDs are of paramount importance. In a short while, these efforts have shown a way to successfully address NCDs and are therefore recommended for adaptation by other nations as an approach to join the global response in tackling NCDs.
